# Supplementary material for: 3D printable diffractive optical elements by liquid immersion
Source: Nat Commun. 2021 May 24;12:3067. doi: 10.1038/s41467-021-23279-6 (PMC8144415; doi:10.1038/s41467-021-23279-6)
Supplement: Supplementary file 1 — Supplementary Information [file 41467_2021_23279_MOESM1_ESM.pdf]

Supplementary Information for:

3D printable diffractive optical elements by liquid immersion

Authors:

Reut Orange-Kedem [1,2], Elias Nehme[2,3], Lucien E. Weiss[2,4], Boris Ferdman[1,2], Onit Alalouf[2,4], Nadav Opatovski[1,2], and Yoav Shechtman[1,2,4*]

[1]Russell Berrie Nanotechnology Institute, Technion - Israel Institute of Technology, Haifa 32000, Israel.

[2]Lorry Lokey Interdisciplinary Center for Life Sciences and Engineering, Technion - Israel Institute of Technology, Haifa 32000, Israel.

[3]Department of Electrical Engineering, Technion - Israel Institute of Technology, Haifa 32000, Israel.

[4]Department of Biomedical Engineering, Technion - Israel Institute of Technology, Haifa 32000, Israel.

*Corresponding author. E-mail: yoavsh@bm.technion.ac.il

Table of Contents

1. Phase mask design
2. The fabrication process of the immersion phase mask
3. Phase mask characterization
4. Choosing a fabrication method
5. Supplementary videos

Note 1. Phase mask design:

The diameter of the phase mask was adjusted to match the back focal plane (BFP) size of the optical setup using the following equation:

$$Phase\ mask\ diameter = \frac{2f \cdot n_{sample}}{\sqrt{M^2 - NA^2}} = 5.3\ mm \quad (S1)$$

Where f is the focal length of the Fourier-transform lens (200 mm), n_{sample} is the refractive index of the sample (1.33), M is the magnification (100), and NA is the numerical aperture (1.35).

To design the phase mask, the target phase map needs to be converted to physical parameters. These include (1) the axial step size; (2) the number of axial steps; (3) the lateral pixel size; (4) the refractive index of the mask material; (5) the refractive index of the immersion liquid; and (5) the emission wavelength. Notably, these parameters are optimized for a printing method, and are described in Supplementary Table 1. One advantage of our scaled up approach is that a larger number of axial steps can be used, effectively leading to a smoother phase function that better resembles the desired mask (Supplementary Figure 1).

Supplementary Table 1. **Phase-mask-design parameters**

Axial step size	5 µm
Number of axial steps	55 (Tetrapod), 57 (Double helix)
Lateral pixel size	20 µm
Mask refractive Index, $\lambda = 668\ nm$	1.42830 (PDMS)
Immersion liquid refractive index, $\lambda = 668\ nm$	1.43060 (Glycerol-water mixture ~72:28)
Emission wavelength	668 nm

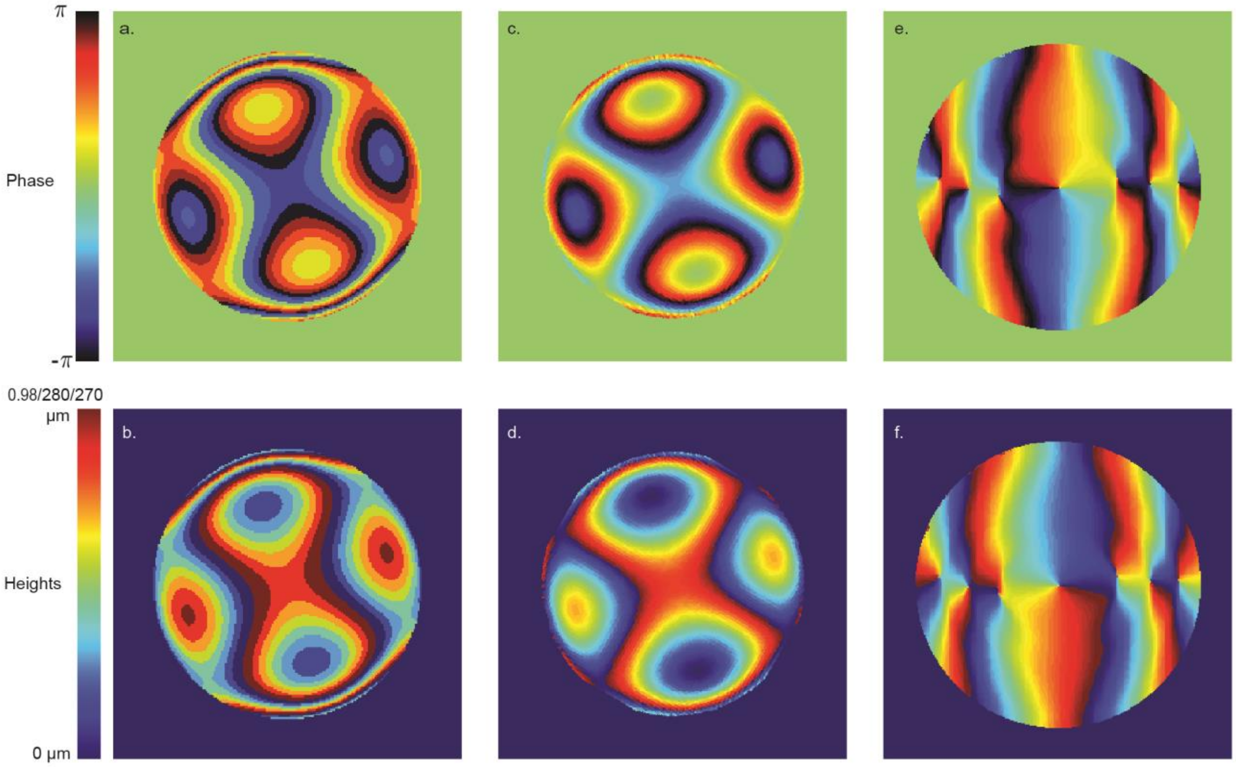

Supplementary Figure 1**. Mask design. a & b** The accumulated phase and height maps of a conventional, photolithographically fabricated Tetrapod phase mask with 8 different heights (step size 140 nm). **c & d** The maps for the liquid-immersed Tetrapod phase mask with 57 heights (step size 5 μm). **e & f** The maps for the liquid immersed Double Helix phase mask with 55 different heights.

Note 2. The Fabrication process of the immersion phase mask

The fabrication process can be done in standard conditions with the equipment listed in Supplementary Table 2, does not require a clean room, and is described below.

1. <u>Fabricating a microscale mold:</u> A ceramic mold (Zirconia) that contains the reverse pattern of the desired phase mask was printed via additive manufacturing (Carmel 1400, Xjet Ltd., Israel). An illustration is shown in Supplementary Figure 2a.
2. <u>Transferring the mold pattern to transparent material:</u> The ceramic mold was first coated with a thin layer of oil (WD40) to reduce adhesion. Next, Polydimethylsiloxane (PDMS, SYLGARD 184, POLYMER G) was prepared by mixing the two-component elastomer and degassing in a vacuum chamber for ~30 minutes to remove bubbles. The PDMS was then poured onto the lubricated mold, and placed into the vacuum chamber to facilitate additional bubble removal. The PDMS was allowed to cure for 24 hours at room temperature on a leveled surface to ensure flatness. The polymerized PDMS was then carefully separated from the ceramic mold, and the outside edges were cleaned with a razorblade. An illustration is shown in Supplementary Figure 2b.
3. <u>Creating the PDMS frame:</u> To create a mold for the outer chamber wall, we machined a metal disk with a slot to hold the liquid-exchange tubes. PDMS was poured on top of the

mold holding the liquid exchange tube against a petri dish, and the assembly was debubbled, and cured as described previously. After 24 hours, the PDMS frame was separated from the metal disk with the tube embedded in the polymer. The tube was then cut at both inner edges of the wall, creating an inlet and outlet for the chamber. An illustration is shown in Supplementary Figure 2c.

4. Assembling the liquid-immersed phase mask: A brief oxygen plasma treatment to PDMS and SiO_2 enables a sturdy, chemical attachment (~1 min per treatment, Zepto W6, Diener Electronic). This process was used to bond the phase mask and outer frame to one fused-silica wafer. Next, the chamber was closed by attaching the second wafer to the frame using the same process. Lastly, an M5 metal washer, with an inside diameter matching the phase mask diameter (5.3 mm), was glued to the external side of fused silica wafer. This reduced the effect of light that might traverse the optical system at spatial frequencies beyond the desired range, *e.g.* supercritical-angle fluorescence. An illustration is shown in Supplementary Figure 2d-f.
5. Adding immersion liquid to the chamber: The device is designed to contain a liquid with refractive index 1.4283 at 668 nm that must interface directly with the PDMS mask, index 1.4306 at 668 nm. To minimize bubbles and voids created while filling the device, we first degas the solution in a vacuum chamber for 5 minutes and prefill the chamber with isopropanol. The glycerol-water mixture is then injected into the chamber replacing the isopropanol. To compensate for any error in the RI of the PDMS or height scaling, the RI of the liquid was tuned iteratively, adjusting the ratio of glycerol and water and evaluating the optical performances of the system at each step by comparison to simulations of the desired 3D-PSF response.
6. Positioning the mask in the optical system: Our device is larger than a typical dielectric phase mask, but nonetheless requires a similar alignment procedure, namely placing the phase mask in the back focal plane. For fine alignment, we attached a threaded adapter to the assembly (SM1S10, Thorlabs) with optical adhesive (NOA68T, Norland Products) and mounted it on a 6-axis kinematic optic mount (Thorlabs K6XS) while monitoring the 3D response of the PSF.

Supplementary Table 2. **Instruments used in device fabrication and characterization**

Instrument	Model Number	Manufacturer	Purpose
Vacuum chamber	402020	Tarson	Degassing PDMS
Plasma cleaner	Zepto W6	Diener Electronic	Bonding PDMS to silica wafers
Refractometer	DR6200TF	A.Krüss Optronic	Characterizing immersion liquid

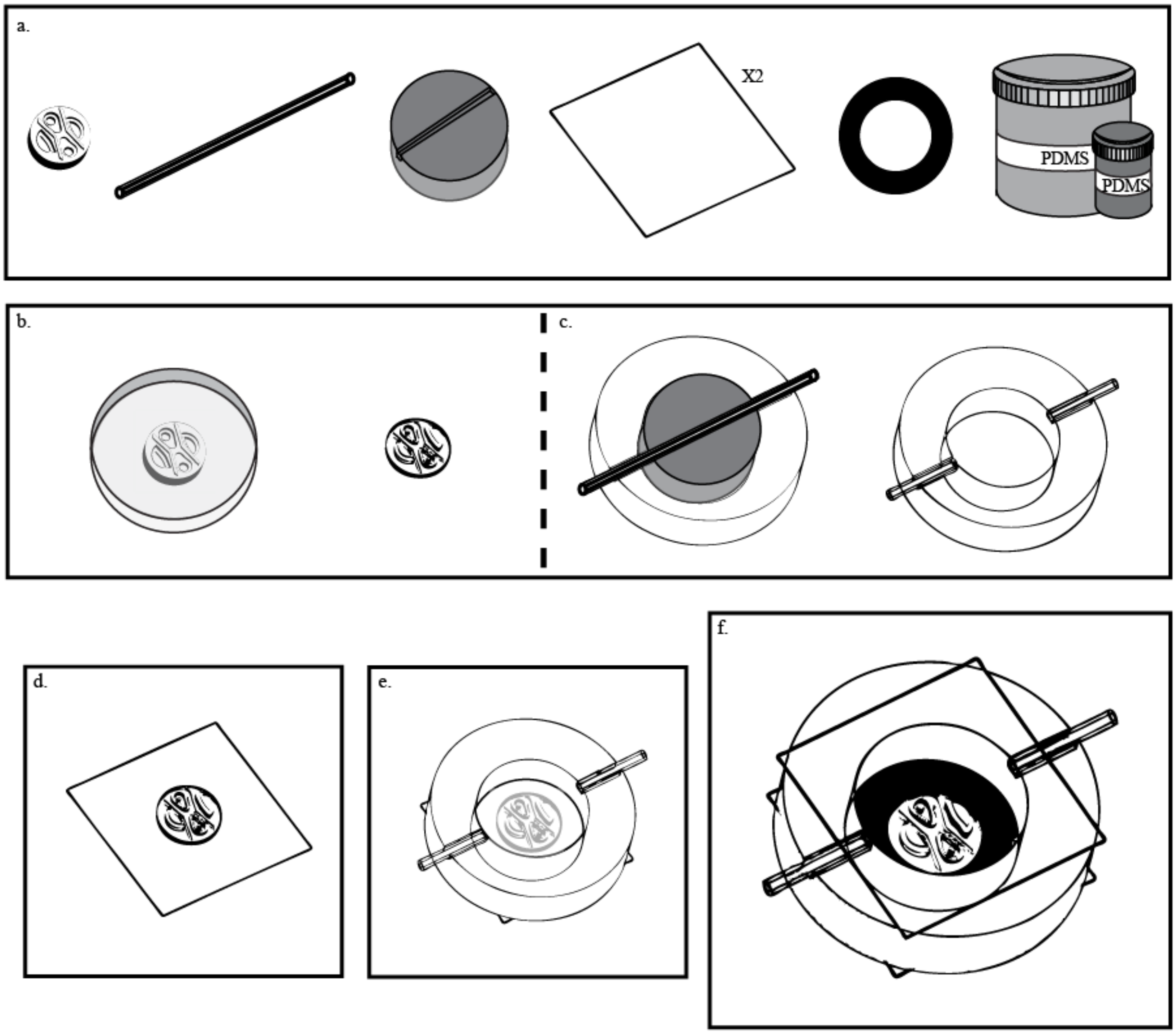

Supplementary Figure 2. **Liquid immersed phase mask fabrication process. a** The components needed to fabricate the mask left to right: a reversed template, a tube (inside diameter ~1mm), metal disk with slot that matches the outer diameter of the tube, two fused silica wafers (or other high optical quality wafers), metal ring matched to the diameter of the mask and PDMS. Preparation steps: **b** polymerization of the PDMS on the mask template and the extracted PDMS mask **c** polymerization of the PDMS on the tubes and metal ring with the slot and the extracted PDMS frame **d** First step: attaching the PDMS mask to the fused silica wafer **e** Second step: attaching PDMS frame to the fused silica wafer **f** attaching the second fused silica wafer to the exposed side of the PDMS and gluing the blocking metal ring with UV adhesive.

Note 3. Phase mask characterization:

1. **Photon efficiency:**

For comparing the photon efficiency of various DOEs, we utilized a simple optical setup shown in Supplementary Figure 3c, where the first lens position is used to add a quadratic phase that approximates the effect of significant defocus. For each mask, we acquired and averaged 6 images. To calculate the attenuation of each mask, we integrated the signal over a small area containing the main PSF. Interestingly, we found that the liquid-immersed mask had a similar photon efficiency relative to the photolithographically fabricated phase mask (integrated counts of 9,544 and 9,869, respectively for the same area in the background subtracted images shown in Supplementary Figure 3 a & b).

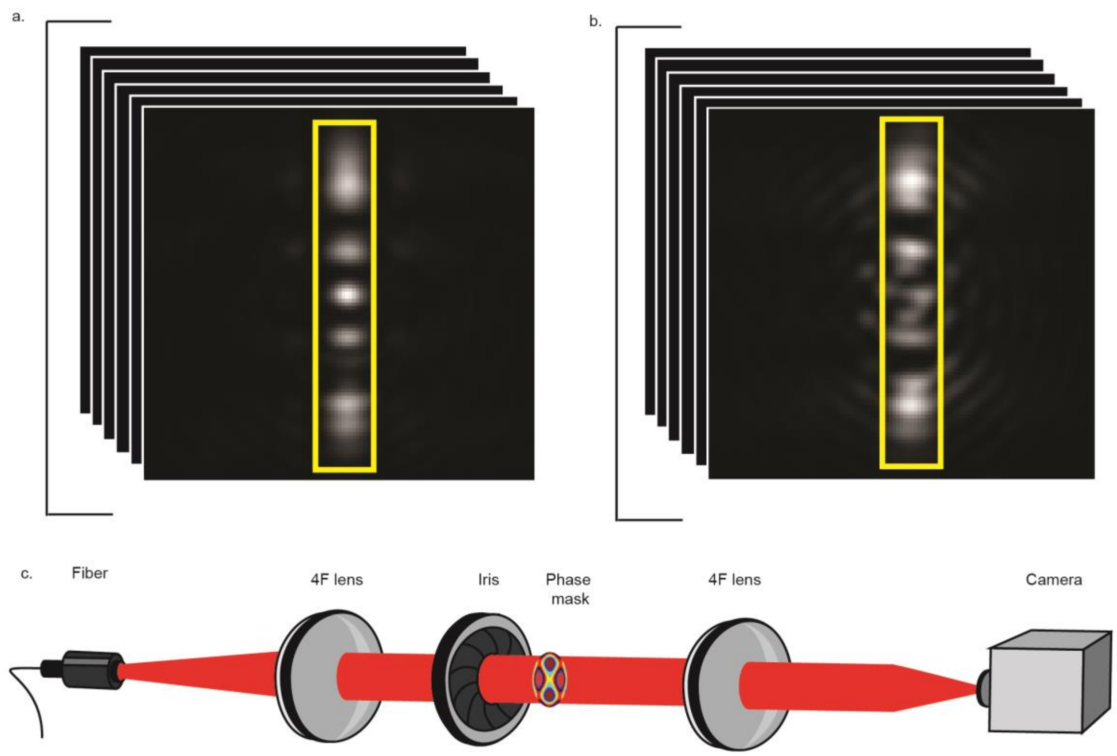

Supplementary Figure 3. **Efficiency test. a** The averaging of 6 images of the modified PSF obtained on the image plane from the photolithographically fabricated Tetrapod (liquid immersed Tetrapod phase mask) resulting from the same parabolic phase. **b** The averaging of 6 images of the modified PSF obtained on the image plane from the liquid immersed Tetrapod phase mask resulting from the same parabolic phase. The yellow rectangles show the relevant area for calculating the intensity as a result of the passage through the masks **c** The optical setup, using a laser as a light source, 4F system (lenses f=20 cm), a phase mask, iris for blocking the light beyond the mask diameter (lenses f=20 cm) and a camera (Pixelink-Pl-D7512MU-T).

2. **Wavefront characterization by off-axis holography:**

To measure the phase pattern produced by the liquid-immersed phase mask and compare it to the desired design, we used off-axis digital holography, which recovers the wavefront of a

beam by backpropagation of a captured interference pattern. The optical setup is illustrated in Supplementary Figure 4, a. The principle of off-axis holography is to capture an interference pattern rather than an image. This is done by splitting a collimated, coherent beam into two parts, namely an object beam and a reference beam, passing the object beam through the phase mask and then overlaying the beams at the sensor plane so that the interference pattern appears

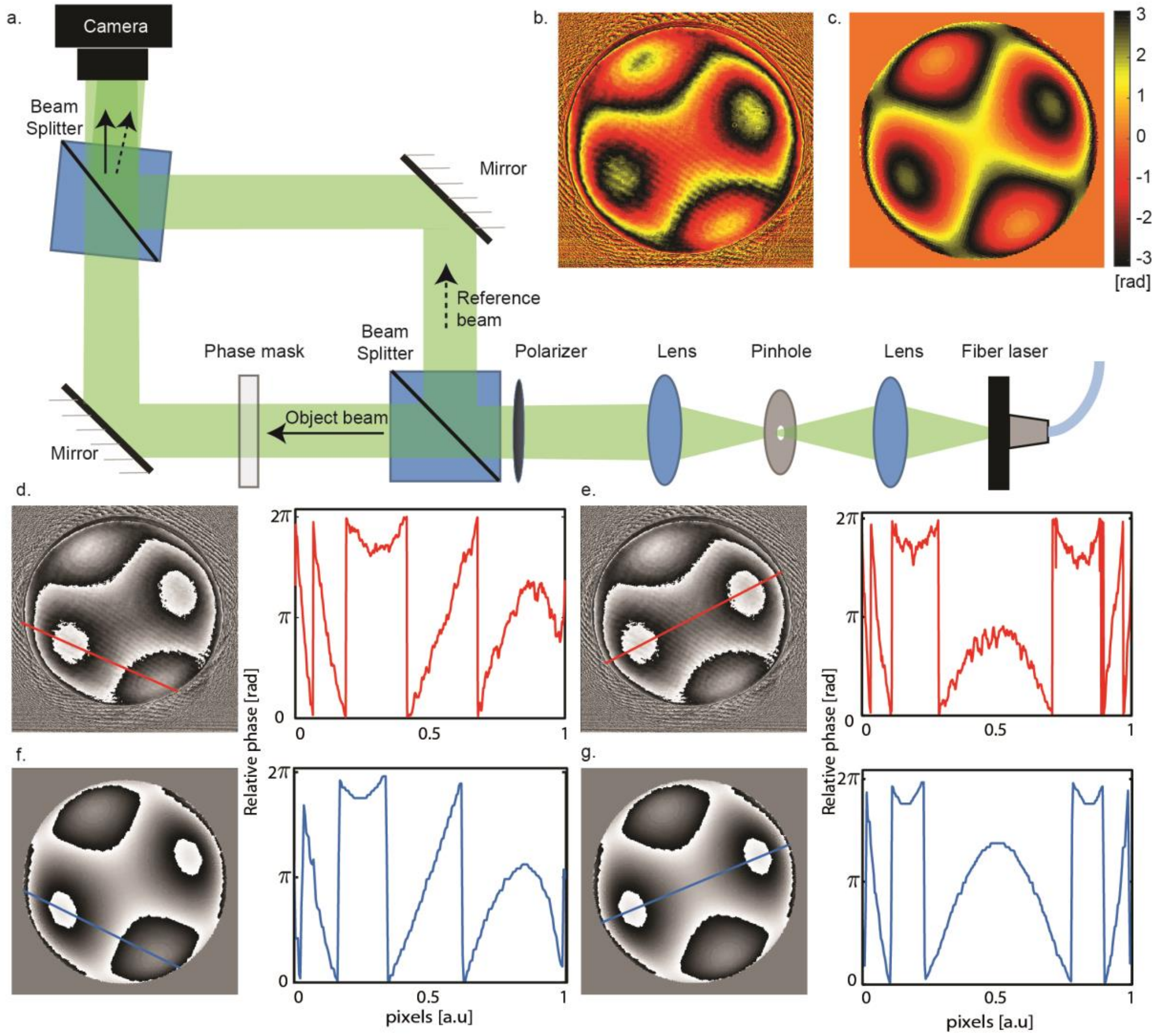

Supplementary Figure 4. **Wavefront characterization by off-axis holography. a** Illustration of the off-axis digital holography setup for phase imaging. A laser beam is first spatially cleaned and collimated. The collimated beam is then split to an object and a reference beam. The object beam goes through the phase mask while the reference beam maintains its smooth wavefront, and both are recombined in an off-axis configuration to generate interference on the camera sensor. **b** The reconstructed wavefront of the liquid immersed phase mask with the following parameters: Δn=0.0018, $\lambda = 514$. **c** The corresponding design of the liquid immersed phase mask. Profile plot of the relative phase of: **d** Side line of the reconstructed mask presented in b. **e** Center line of the reconstructed mask presented in b. **f** Side line of the design presented in c. **g** Center line of the design presented in c.

on the sensor. The captured interference intensity is described in Equation S2:

$$I(x,y) = |R|^2 + |O|^2 + (RO^* + OR^*) \quad \text{S2}$$

Where R, O stand for the complex wavefronts of the reference and object beams, respectively. * denotes the complex conjugate operation. The term in the brackets is real-valued but contains the phase of the object beam, which is absent in a regular, intensity-only, imaging apparatus. The presence of the bracketed term provides information on the object beam wavefront at the sensor plane, which is enough to back-propagate it for reconstruction at any other plane. The mathematical process of reconstruction is described in detail by N. Verrier and M. Atlan[1].

3. Signal transmission through the device interfaces:

The wafers in our liquid-immersion phase mask are high-quality fused silica, note that the same material was also used in our photolithography-based fabrication of phase masks. The transmission of the device is hampered mainly by reflections; however, since most reflections are between layers with very close refractive indices, the overall effect on transmittance is small, as detailed below.

According to Fresnel-coefficients equations, under the assumptions that (1) the incident angel of the wavefront is near 0, and (2) the transition between the PDMS to the liquid does add a reflection, we can calculate the power transmittance, T, following equations S3 and S4 noting that both polarization coefficients are equal due to assumption (1):

$$t = \frac{2n_1}{n_1+n_2}, \quad \text{S3}$$

$$T_{single\ polizarization} = \frac{1}{2}\frac{n_2}{n_1}|t|^2 \rightarrow T_{both\ polizarizations} = \frac{n_2}{n_1}|t|^2 , \quad \text{S4}$$

where t is the amplitude transmission coefficient of the electric field. The mask structure is described in Supplementary Figure 5.

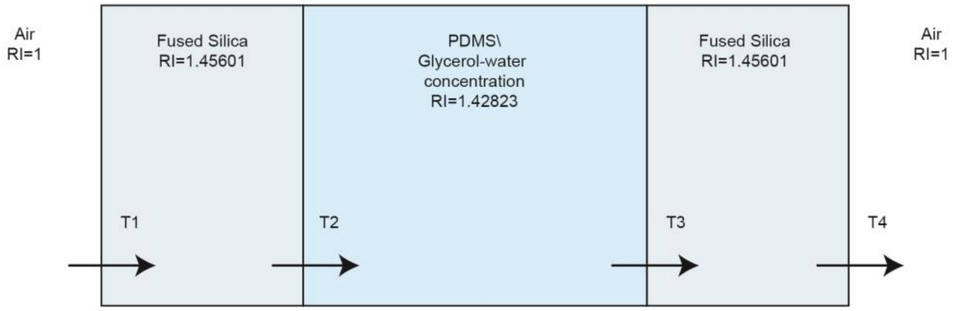

Supplementary Figure 5. **Simplified model of the liquid-immersed chamber**. specifying the different layers and the corresponded refractive indices. T1, T2, T3, T4 are the power transmittance between the different interfaces.

The total power transmittance is the product of all transition terms, T. For the liquid immersed phase mask, $T_{total} = T_1 \cdot T_2 \cdot T_3 \cdot T_4$= 0.9312. For the photolithographically fabricated phase mask $T_{total} = T_1 \cdot T_4$ =0.9313. Namely, the additional reflections at the interfaces lead to a signal reduction of 0.01%.

Notably, the most significant contributors to the total reflectance are the transitions with the largest refractive index changes, i.e. from air to fused silica ($T_1 = 0.9650$), and Fused Silica to air ($T_4 = 0.9650$), leading to a total reduction of ~7%. Of course, these transitions are also present for both phase mask types, but could be further reduced with antireflective coatings.

4. Linear-phase estimation:

Although the assembly of the device was not done with specialized equipment, we did not detect significant aberrations in the PSF relative to the design. We used high quality fused silica wafers (surface roughness: Ra< 1 nm, surface quality: 20/10 scratch-dig number, total thickness variation <3.5 µm), so the wafers should not add any unexpected aberrations. One potential assembly error of our bench-top device is in the non-parallelism in the chamber (illustrated in Supplementary Figure 6), we measured the height differential across the device to be <0.2°, and < 0.1° across the printed-mask mold. The result of this slight height change across the device would manifest itself in a small linear phase accumulation that would shift the beam path.

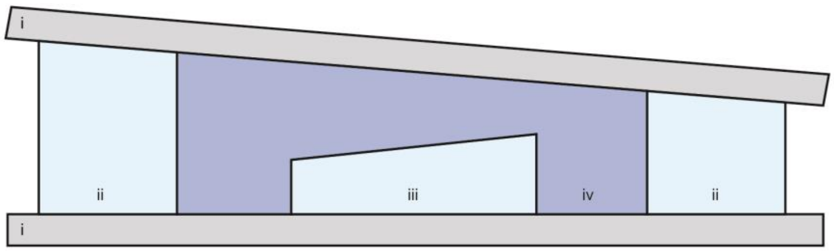

Supplementary Figure 6. **An illustration of a possible deviation in parallelism of the different planes of the phase mask assembly (tilt is extremely exaggerated for clarity). i** the two silica wafers **ii** The PDMS frame **iii** The PDMS mask **iv** The liquid. The deviation in parallelism can causes from deviation in the printed mask which leads to deviation in the PDMS mask or from the PDMS frame polymerization.

Note 4. Choosing a fabrication method

A brief explanation of the key considerations that factor into the choice of printing methods for a mask design are described below.

<u>Phase mask suitability:</u>

In this work, we used the Xjet printer, which has a maximum height change of 150 µm per 40 µm lateral change. This maximal aspect ratio proved to be sufficient for the DOEs we demonstrate, however there can be more challenging DOEs, e.g. optical gratings, where this could pose a limitation.

The effect of scaling up mask dimensions:

As we reduce the refractive index difference, the heights of the mask should increase. Then the second term from equation S5, becomes more significant.

$$\delta\Delta\phi_{\mathrm{Error}} \approx \frac{2\pi}{\lambda}(\Delta n \cdot \delta h + h \cdot \delta n) \quad \text{S5}$$

To estimate the tolerance in fabrication error at a specific refractive index difference between the mask and the surrounding media, we first calculate the $\delta\Delta\phi_{\mathrm{Error}}$ of the photolithography phase mask. The mean accumulated fabrication error measured in our photolithography fabrication was ~70 nm. With a $\Delta n \sim 0.5$, $\lambda = 668\ nm$, we can calculate the relative phase error to be $\delta\Delta\phi_{\mathrm{Error}} \approx \frac{2\pi \cdot 0.5 \cdot 0.07}{0.668} = 0.3292$ radians.

Now, we can estimate the fabrication precision of the current method required to achieve the same level of phase error. Here, $\Delta n = 0.0023, h_{\mathrm{mean}} = 140\ \mu\mathrm{m}$. Assuming the precision of the glycerol-water concentration to be $\sim 1 \times 10^{-5}$, namely the precision of the refractometer, the fabrication error which would yield the same fabrication error as the photolithography case can be calculated to be:

$$0.3292 \text{ radians} \approx \frac{2\pi}{\lambda}(\Delta n \cdot \delta h + h \cdot \delta n) \quad \text{S6}$$

$$\delta h = \frac{0.5 \cdot 0.07\ \mu\mathrm{m} - 140\ \mu\mathrm{m} \cdot 0.00001}{0.0023} = 14.61 \mu\mathrm{m} \quad \text{S7}$$

This parameter provides an estimation of the fabrication precision needed.

Fabrication precision:

When printing a template for phase mask fabrication, the key parameter is the relative error, i.e. the deviation from the upper profile of the printed part. While the precise error is difficult to quantify, the fabrication error for the instrument is quoted to be better than 50 µm in the axial and lateral directions. Nevertheless, we estimate the axial precision, which is the most important dimension in this application, to be closer to ~15 µm or less. This is because, in practice, our mask's performance is similar to a photolithographically fabricated mask, and this is the axial precision required to obtain such results (see SI section IV). Notably, some fabrication error, specifically a vertical scaling in the mask, can be corrected for with the liquid immersion calibration process.

Immersion-media characterization:

A concentration error of the immersion media results in a change in the refractive index. To monitor the mask-immersion liquid in the device, we characterized the refractive index with a commercial refractometer (DR6200TF, A. Krüss Optronic, GmbH), precision $\pm 1 \times 10^{-5}$. In practice, tuning the immersion media to a less precise extent $\pm 1 \times 10^{-4}$ was sufficient for our purposes.

Note5. Supplementary movies descriptions:

Supplementary movie 1: **Super-resolution reconstruction of mitochondria**. 3D rendering of mitochondria spanning a 3.6-μm axial range, corresponding to main text Figure 3a. Scale bar 5 μm.

Supplementary movie 2: **Super-resolution reconstruction of microtubules.** 3D rendering of the super-resolved microtubules spanning a 2.8-μm axial range, corresponding to main text Figure 3b. Scale bar 5 μm.

Supplementary references:

1. Verrier N, Atlan M. Off-axis digital hologram reconstruction: some practical considerations. *Appl Opt*. 2011;50(34):H136. doi:10.1364/AO.50.00H136
