# Supplementary material for: 3D printable diffractive optical elements by liquid immersion
Source: Nat Commun. 2021 May 24;12:3067. doi: 10.1038/s41467-021-23279-6 (PMC8144415; doi:10.1038/s41467-021-23279-6)
Supplement: Supplementary file 3 — Description of Additional Supplementary Files [file 41467_2021_23279_MOESM3_ESM.pdf]

## Description of Additional Supplementary Files

Supplementary Movie 1: Super-resolution reconstruction of mitochondria

Supplementary Movie 2: Super-resolution reconstruction of microtubules
