# Supplementary figures and images for: 3D printable diffractive optical elements by liquid immersion
Source: Nat Commun. 2021 May 24;12:3067. doi: 10.1038/s41467-021-23279-6 (PMC8144415; doi:10.1038/s41467-021-23279-6)

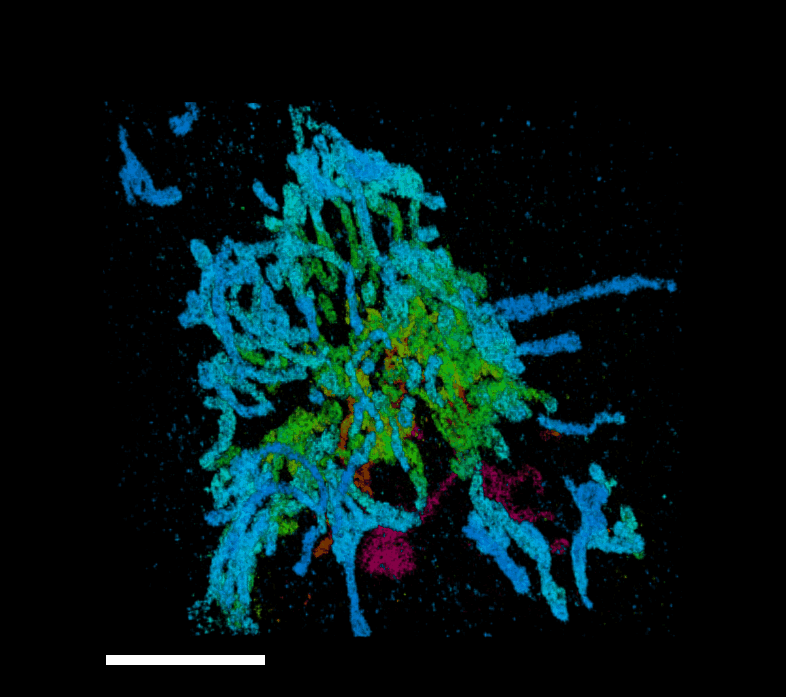

Supplement: Supplementary file 4 — Supplementary Movie 1 [file 41467_2021_23279_MOESM4_ESM.gif]

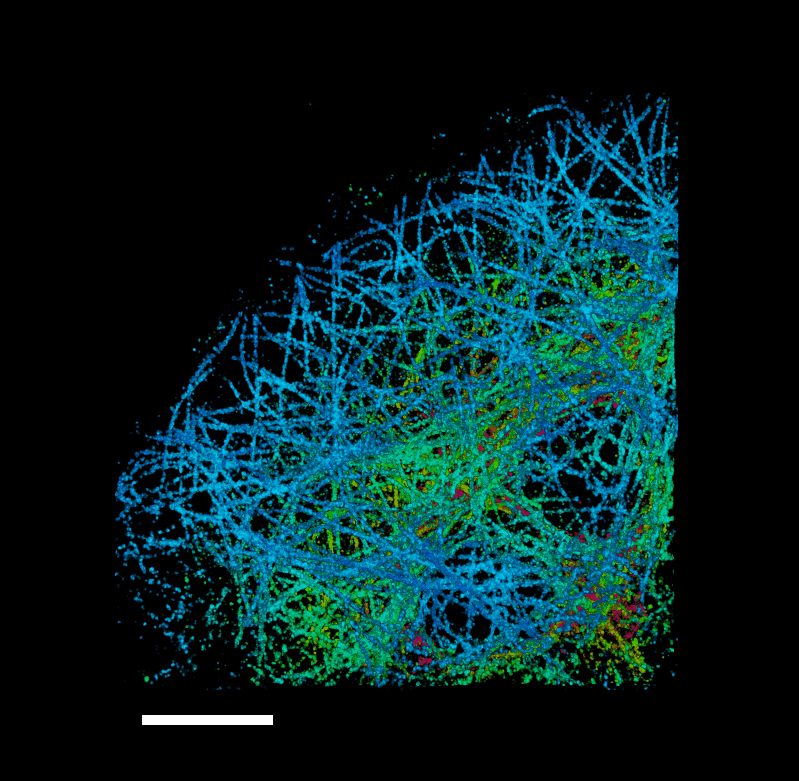

Supplement: Supplementary file 5 — Supplementary Movie 2 [file 41467_2021_23279_MOESM5_ESM.gif]
